# Comorbidity in primary care – causal or casual? A longitudinal observational study in family medicine

**DOI:** 10.1186/s12875-024-02513-2

**Published:** 2024-08-02

**Authors:** Jean K. Soler, Nicola Buono, Inge Okkes

**Affiliations:** 1 Mediterranean Institute of Primary Care, 19, Triq ir-Rand, Attard, ATD1300 Malta; 2 MD, ICPC Club, Caserta, Piazza Matteotti 67 81100 Italia; 3 Department of General Practice, Academic Medical Centre, University of Amsterdam, Amsterdam, The Netherlands

**Keywords:** Comorbidity, Causality, International classification of primary care, Family practice, General practice, Primary care, Episode of care, Electronic medical record, The Netherlands, Malta, Serbia

## Abstract

**Background:**

Comorbidity is increasingly important in the medical literature, with ever-increasing implications for diagnosis, treatment, prognosis, management and health care. The objective of this study is to measure casual versus causal comorbidity in primary care in three family practice populations.

**Methods:**

This is a longitudinal observational study using the Transition Project datasets. Transition Project family doctors in the Netherlands, Malta and Serbia recorded details of all patient contacts in an episode of care structure using electronic medical records and the International Classification of Primary Care, collecting data on all elements of the doctor-patient encounter, including diagnoses (1,178,178 in the Netherlands, 93,606 in Malta, 405,150 in Serbia), observing 158,370 patient years in the Netherlands, 43,577 in Malta, 72,673 in Serbia. Comorbidity was measured using the odds ratio of both conditions being incident or rest-prevalent in the same patient in one-year dataframes, as against not, corrected for the prior probability of such co-occurrence, between the 41 joint most prevalent (joint top 20) episode titles in the three populations. Specific associations were explored in different age groups to observe the changes in odds ratios with increasing age as a surrogate for a temporal or biological gradient.

**Results:**

The high frequency of observed comorbidity with low consistency in both clinically and statistically significant odds ratios across populations indicates more casual than causal associations. A causal relationship would be expected to be manifest more consistently across populations. Even in the minority of cases where odds ratios were consistent between countries and numerically larger, those associations were observed to weaken with increasing patient age.

**Conclusion:**

After applying accepted criteria for testing the causality of associations, most observed primary care comorbidity is due to chance, likely as a result of increasing illness diversity.

**Trial registration:**

This study was performed on electronic patient record datasets made publicly available by the University of Amsterdam Department of General Practice, and did not involve any patient intervention.

**Supplementary Information:**

The online version contains supplementary material available at 10.1186/s12875-024-02513-2.

## Key points

Comorbidity is very commonly observed in primary care patients.

Most of the comorbidity observed in primary care is due to chance, and causal relationships between co-occurring diseases are uncommon.

Most observed comorbidity may be an artefact of increasing illness diversity.

Comorbidity studies require appropriate classifications and episode of care models.

**Supplementary Information:**

The online version contains supplementary material available at 10.1186/s12875-024-02513-2.

## Background

Comorbidity is increasingly important in the medical literature, with ever-increasing impacts as populations age. Comorbidity has multiple and complex implications for the processes of diagnosis (one condition increases the risk of having another), treatment (more than one disease must be treated concurrently), prognosis, management and health care [1]. Yet, the term itself, as well as related terms such as multimorbidity and morbidity burden, may be inconsistently conceptualised [1].

Comorbidity is broadly defined as the co-occurrence of two diseases in the same patient. However it is most often defined in relation to an index condition, and often, but not exclusively, at the same time [1]. Two unrelated medical conditions can co-occur due to chance, but one disease can also cause another, or increase the risk of another developing or worsening [1]. However, how does one reliably distinguish between causal and casual relationships, and above all, what proportion of associations are simply casual? In 1965, Sir Austin Bradford Hill’s presidential address to the Royal Society of Medicine addressed this issue, and suggested nine criteria for judging an association to be causal rather than casual. These were: strength of association, consistency (across different studies or populations), specificity, temporal relationship, biological gradient (or dose–response), plausibility, coherence, experimental evidence and analogy [2]. These criteria are still used today in modified form. It is interesting that strength and consistency of association were the first two criteria Sir Bradford Hill presented.

Anxieties about missing an important diagnosis or under-treating dangerous morbidity may cause clinicians to over-estimate the relevance of relationships between casually comorbid conditions. Guidelines may decrease the threshold for the diagnosis of one disease given the presence of another (for example, a lowered threshold for diagnosing hypertension in diabetic patients). Surveillance for one disease may increase the chance of finding another, as would repeat doctor-patient encounters once a chronic disease is diagnosed. In fact, the strength of an apparent comorbid association may weaken or disappear once one adjusts for the number of health care contacts. A classic example is the probably spurious association between depression and diabetes [3]. As such, it is important to collect data in an episode of care (EoC) model which captures distinct episodes over time. Additionally, the analysis of the causality of an apparent association is improved by correcting the observed association for the prior probability of co-occurrence due to chance [1].

The Transition Project international family practice databases are an international standard presentation [4–7]. The data represent unique perspectives on day to day usual family practice care, on all problems managed, are longitudinal, are organised using an episode of care data structure and are coded with the International Classification of Primary Care (ICPC) [8]. They provide opportunity to address the following objective and answer the following questions.

### Objective

The objective of this study is to measure casual versus causal comorbidity in primary care as observed in three family practice populations.

### Research questions

- What is the observed comorbidity of the 20 jointly most common episodes of care in three countries?
- How much of the observed comorbidity is likely to be casual versus causal?

## Materials and methods

### Setting

The Transition Project databases contain data on each encounter in family practices in the Netherlands, Malta and Serbia over time [4–7], including almost 1.7 million diagnoses over 275,000 patient years’ observation.

### Population and databases

The practice populations in the Netherlands and Serbia represent registered patient populations: 158,370 patient years (1995 to 2005) and 72,673 patients (over 15 years of age) in one year (2003) respectively. The population in Malta represents patients consulting over a five-year period from 2001 to 2005 (43,577 patient years). The databases were analysed using a one-year data-frame, starting on the 1st January of each year of observation. An EoC open over a number of years of observation would be re-coded as rest-prevalent (to distinguish it from incident/new) in subsequent years, as in our previous studies of incidence and prevalence [4–7].

### Data

The public-domain electronic medical record (EMR) “TransHis” [4], designed for use with ICPC [8], was used to collect data from participating family doctors (FDs) who recorded elements (reason/s for encounter, diagnosis/es and intervention/s) of all their primary care patient contacts in an EoC structure using ICPC. For this study, the diagnostic labels of the episode of care (*episode titles*) were analysed [4–7].

An *episode of care* (EoC) is defined as a health problem from its first presentation by the patient to the FD, until the completion of the last encounter for it. Its label (i.e. the *episode title,* the diagnostic label of the EoC) may be modified over time to reflect a change in the clinical picture [8]*.*

### Analysis

Comorbidity was defined as *all other episodes of care co-existing with an episode of care in a defined time period,* that time period being one calendar year data-frame [4]. The odds ratio (OR) for a comorbidity is the ratio of the probability of occurrence of the comorbidity to that of non-occurrence. It takes into account that the posterior probability of a diagnosis given another not only depends on the extent to which the two diagnoses are associated, but also on the frequency of the disease/episode of care in the practice population (the prior probability). The probability of the disease/episode of care given the comorbidity is the posterior probability, which is expressed as an OR [4].

The databases were used to study the OR of one specific EoC being incident or rest-prevalent in a patient, given the incidence or rest-prevalence of another specific EoC in the same patient, as against not, in a one-year dataframe, with a 95% confidence interval (calculated according to Altman et al.) [4, 9]. The joint common top 20 distribution of EoCs in the three countries was used for this study, to limit the number of contrasts to the most frequent EoCs. Forty-one episode titles described the top 20 most prevalent EoCs in all three countries jointly [6].

In selected cases exhibiting high ORs (i.e. strong observed comorbidity) across multiple databases, we attempted to further study the causal versus casual nature of such associations by analysing comorbidity across age groups. One would generally expect diseases to have likely been prevalent for longer in older patients. Thus the disease would likely have had more time in which to have an effect on the individual patient as well as the other index disease. Thus, any causal effect would be reasonably expected to be more evident. The expected effect of one disease being present over a longer period of time (in older patients) and of having a causal relationship with the other, would be that the OR would rise (or an OR less than 1 would be expected to fall) in older age groups.

Age was calculated at the middle of the observation frame [4–7].

### Clinical and statistical significance

The minimum level of *clinical significance* for an OR was arbitrarily taken as that which represents a standardised difference of at least 0·10 (10% of the variability is so explained). This is equivalent to a relative risk of 2·0 or more [7, 9]. Since the OR tends to overestimate the relative risk, an arbitrary cut-off level of > 2 for the OR of a positive association, and < 0·5 for the OR of a negative association, were taken as thresholds for clinical significance. An OR of > 8 or < 0·2 was classified as a strong predictor [10]. Furthermore, ORs which are not at least as large as their confidence interval (CI) were arbitrarily ignored as unreliable [7, 10].

### Ethical approval

The study did not involve the collection of new data. Ethical approval was applied for locally, when appropriate, for the original data collection and for individual studies based on these data in the Netherlands, Malta and Serbia.

## Results

We would suggest referring to a printed copy of all ICPC rubrics and short text labels whilst reading the results and discussion sections (Appendix 1).

### Distribution of comorbidity

Table S1 in Supplementary Material 2 gives ORs of one specific EoC being incident or rest-prevalent in the one year period, given the incidence or rest-prevalence of another specific EoC in the same patient, as against not, with a 95% confidence interval. Forty-one episode titles described the top 20 most prevalent EoCs in all three countries jointly. Clinically and statistically significant ORs are highlighted (see table key) and those which are clinically and statistically significant in all three databases are highlighted in yellow. The large number of significant ORs is immediately evident, demonstrating the high frequency of observed comorbidity in primary care.

Table 1 is a summary of the clinically and statistically significant relationships in Table S1 in Supplementary Material 2. Of the 820 sets of analyses (each row of three associations between two EoCs, one from each of the three databases, comprising one set), 573 (69·9%) showed at least one significant association from at least one of the three databases. However, a consistently significant set of ORs (i.e. significant in all three databases) was observed in only 76 (9·3%) cases. Only 32 (3·9%) sets contained at least one significant OR which was a strong association. There was no single set with strong associations consistently present in all three databases. However in 7 cases (0·9%), a set of three consistent associations contained two strong ones (data not tabulated, see below). Significant inverse associations (OR significantly less than 1) were rare, being found in only 6 (0·7%) sets of comparisons, and in only 2 (0·2%) cases was at least one of these inverse associations strong. Again, there were no cases of consistently significant inverse associations, consistent across all three databases. Additionally, we did not observe any set with contrasting significant ORs in differing directions from unity. As such it appears that strong, likely causal, comorbid associations are rare in primary care, and when observed they are not consistent across different populations.

**Table 1:** Summary of the clinically and statistically significant relationships in Table S1 in Supplementary Material 2

ICPC rubrics in common Top 20	41
Number of possible comorbidities (combinations)	820
Number of rows with at least one significant comorbidity	573
Number of rows with three significant comorbidities	76
Number of rows with at least one strong comorbidity	32
Number of rows with three strong comorbidities	0
Number or rows with at least one inverse comorbidity*	6
Number of rows with three inverse comorbidities*	0
Number of rows with at least one strong inverse comorbidity*	2
Number of rows with three strong inverse comorbidities*	0
Numbers of rows at least one contrasting comorbidity†	0

Description and explanation in text. Numbers represent count of rows, representing comorbidities in three country datasets, which have the described characteristics

* i.e. OR less than unity

† Rows with at least one significant OR in the opposite direction of another significant OR

### Extended comparison of comorbidity rates over time (age)

Table 2 is an extended comparison of selected diagnoses and their comorbidity in different age groups. Should a disease have a significant causal relationship with the other, the OR would be expected to increase in older age groups. We arbitrarily selected the cells in Table 1 with three consistent associations (across countries) and with at least two strong associations (ICPC codes K86 *hypertension* and T90 *diabetes type II*; T93 *lipid disorder* and T90; T93 and K74 *ischaemic heart disease with angina*; T93 and K87 *hypertension with end-organ damage*; T90 and K77 *heart failure*; K74 and K77; K77 and K87). The actual observed trend was that of ORs tending to be lower in older age groups (Table 2), suggesting that the dose–response effect is not present, and as such the associations are likely not causal.

**Table 2:**
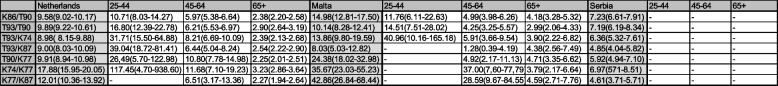
Comorbidity by age group

Comorbidity in different age groups. Odds ratios of selected comorbidities in three age groups (25-44; 45-64; 65+; with first column total population OR for comparison), where associations were consistent across three countries, with at least two being strong

## Discussion

### Research questions

We describe the observed comorbidity of the 20 jointly most common episodes of care in primary care populations from the Netherlands, Malta and Serbia. Out of 820 possible comorbidities, 573 (69·9%) showed at least one significant association from at least one of the three databases. Consistently significant sets of ORs were observed in only 76 (9·3%) cases. Only 32 (3·9%) sets contained at least one strong association. Significant inverse associations were rare (only 6 (0·7%) sets), and in only 2 (0·2%) cases was at least one of these inverse associations strong. We did not observe any set with contrasting significant ORs. Observed comorbidity seems very common, but inconsistent across the country databases, not in direction (negative versus positive) but rather in both strength and significance.

The high frequency of observed comorbidity with low consistency in both clinical and statistical significance between populations indicates more casual than causal associations. A causal relationship would be expected to be manifest more consistently across populations. Even in the minority of cases where the associations were consistent between countries and numerically larger, the associations were observed to weaken with increasing patient age.

### Method and analysis

We defined comorbidity as *all other episodes of care co-existing with an episode of care in a defined time period,* that time period being one calendar year data-frame [4]. Our definition is consistent with most literature [1].

ICPC is a standard instrument to measure the content of primary care, and is an accepted tool to measure comorbidity. In fact, the use of a classification allows more precise measurement of the relationships between unique distinct concepts [1, 4–7]. The granularity of ICPC is appropriate for the study of comorbidity in primary care populations, since the precision of incidence and prevalence estimates is improved with fewer classes (as compared to ICD-10), thus allowing narrower confidence intervals of estimates [11].

The episode of care data model and the practice of coding the symptom diagnosis, when appropriate, keeps disease classes clean, avoids over-estimation of the prevalence of chronic diseases and corrects for the effect of multiple consultations [3–7].

By applying strict limits for clinical and statistical significance one avoids describing spurious associations. The clinical and statistical significance thresholds we used represent a standard presentation which has been extensively validated [5–7, 9] and fits well with Bradford Hill’s requirement for strength of an association being an indicator of causality [2].

We considered Bradford Hill’s criteria for causality [2] and analysed them mathematically. As such our analysis of causality was based on the strength of an association, consistency across different populations, and a temporal relationship. We corrected our ORs for the prior probability, and demonstrated significant departures from the prior probability as recommended by other authors [1]. We then analysed ORs in different age groups as a surrogate for biological gradient or dose–response to re-test our findings. We did not individually assess such associations for specificity, plausibility, coherence, experimental evidence or analogy, partly due to the large number of such associations, and also because our study was not one of individual associations, but rather of general trends.

### Validation through extended comparison

We selected triplets of ORs which were consistently significant across three databases, with two of them strong, for further analysis. In fact, many of such comorbid pairs (K86 and T90; T93 and T90; T93 and K74; T93 and K87; T90 and K77; K74 and K77; K77 and K87) fit established medical literature on important comorbid conditions. We then proceeded to test these selected associations in different age groups.

The Bradford Hill criteria include a dose–response or biological gradient effect [2]. One would expect ORs for a causal association to increase as patients are exposed to the disease/s of interest over time. We used age as a surrogate for the passage of time. We would thus expect the ORs in older age groups to increase for a causal association. We actually observed the opposite, with ORs falling in older age groups.

A lower OR in an (older) age group might alternatively be explained by an increased prior probability of either disease in that age group, and, consequently, also of the comorbidity. However, the OR would not decrease should the posterior probability also increase at the same, or at a larger, rate. This latter case would be expected with a causal relationship exhibiting a dose–effect. As such, this is an unlikely explanation for our findings.

A higher OR for comorbidity may manifest in younger age groups as a consequence of the early presentation of a serious disease which might trigger, in turn, more frequent visits to the FD [3], or increased surveillance or decreased diagnostic thresholds for other related diseases (such as for diagnosing hypertension or hyperlipidaemia in a diabetic). However we have corrected for the effect of multiple visits with the episode of care model, and we have also corrected for the prior probability of such an occurrence.

As such we consider the lack of a dose–response or age gradient as a strong indicator of the lack of a causal relationship. We conclude that the null hypothesis for causality is not rejected.

### Illness diversity

Arguably, the major reason for the large number of significant associations between the commonest EoCs in three populations is *illness diversity*: the increasing number of possible diagnoses in medicine and primary care due to new diagnostic entities. This has the effect of increasing the probability of such interactions. We conclude that most observed associations do not reflect actual causal relationships. Evidently, the utilisation of highly granular coding systems in primary care, especially those which do not separate unique concepts or allow multiple terming of individual concepts, runs the risk of worsening this artefactual comorbid landscape.

### Existing literature

Our approach to studying comorbidity is consistent with prior definitions of comorbidity and with methods to assess both the strength and the clinical and statistical significance of an association [1–3, 9]. Our finding that most comorbidity is likely to be casual may be more controversial. However, such is supported by publications which have sought to confirm the reported causality of specific comorbidities and instead concluded that prior research had failed to adequately correct for chance and/or for the effect of multiple consultations over time [3]. Studies which analysed comorbidity across a range of common health problems and/or different populations and/or captured episodes of care were rare. The Transition Project databases thus provide a unique perspective on comorbidity in primary care.

### Generalisability

FDs are often selected to participate in research using EMRs, and may collect data at a higher level of detail and accuracy than their colleagues. Thus, the analysis of such data sacrifices some generalisability for increased depth, whilst accepting inherent features of family practice which cannot be adjusted for mathematically without introducing new systematic errors and biases. However, we have demonstrated elsewhere that such studies of EMR data are complementary to epidemiological surveys, and are not necessarily less valid or less generalizable [6, 7, 12].

### Limitations

The databases were collected for research purposes from selected practices, with the exception of Serbia. A comparison of data from more practices and more countries, had they been available, would have allowed a more powerful study. A key message is that more data are needed for such comparisons, and this research should be extended to other countries.

### Strengths

The error-trapping and coding support tools in the EMR TransHis, and the advantage of the classification used and the EoC data model have been previously described [4–7]. These qualities are a substantial strength, adding support for the study conclusions.

This is a study of comorbidity which does not focus on a small selection of diseases, but rather analyses data on many common diseases. As such it has significant advantages over studies which either focus on one index disease or collect data from secondary care.

### Impact and future research

This study informs clinicians on the landscape of common comorbidities and allows more rational interpretations, discarding assumed causal relationships and helping to avoid over-treatment. Future research could focus on more sophisticated longitudinal analyses to specifically measure the change of comorbidity ORs over time to quantify the dose–response effect, if present.

## Conclusions

After applying standard criteria for testing the causality of comorbid associations in our study of almost 1.7 million diagnoses over 275,000 patient years’ observation, it is reasonable to conclude that most of the comorbidity frequently observed in primary care is due to chance. Given the lack of strong, consistent associations across different populations and the lack of dose–response effect with age in our study, we conclude that it would be incorrect to assume causal relationships between co-occurring diseases in family medicine as a default position, even if such a relationship might be plausible, or consistent with current conceptualisations of the causation of disease. The study of comorbidity in primary care is most appropriate when using a classification with the appropriate granularity, ordering data according to episodes of care, and with adequate handling of the phenomenon of the symptom diagnosis. Most observed comorbidity in primary care is likely the result of increasing illness diversity.

### Supplementary Information

Supplementary Material 1.

Supplementary Material 2.

## Data Availability

The databases of the Transition Project are publicly available in a CD-ROM included with the ICPC-2-R manual [4] and from the “downloads” section of the website of the Mediterranean Institute of Primary Care (http://www.mipc.org.mt/Downloads.html. Accessed 10^th^ April 2021). The datasets used and/or analysed during the current study are also available from the corresponding author on reasonable request.

## Abbreviations

EMR: Electronic medical records
EoC: Episode of care
FD: Family doctor
ICPC: International Classification of Primary Care
OR: Odds ratio
