# Supplementary material for: Comorbidity in primary care – causal or casual? A longitudinal observational study in family medicine
Source: BMC Prim Care. 2024 Aug 2;25:279. doi: 10.1186/s12875-024-02513-2 (PMC11295527; doi:10.1186/s12875-024-02513-2)
Supplement: Supplementary file 1 — Supplementary Material 1. [file 12875_2024_2513_MOESM1_ESM.pdf]

ICPC-2 – English

International Classification of Primary Care – 2nd Edition

Wonca International Classification Committee (WICC)

Process codes

-30	Medical Exam/Eval-Complete
-31	Medical Examination/Health Evaluation-Partial/Pre-op check
-32	Sensitivity Test
-33	Microbiological/Immunological Test
-34	Blood Test
-35	Urine Test
-36	Faeces Test
-37	Histological/Exfoliative Cytology
-38	Other Laboratory Test NEC
-39	Physical Function Test
-40	Diagnostic Endoscopy
-41	Diagnostic Radiology/Imaging
-42	Electrical Tracings
-43	Other Diagnostic Procedures
-44	Preventive Imunisations/Medications
-45	Observe/Educate/Advice/Diet
-46	Consult with Primary Care Provider
-47	Consultation with Specialist
-48	Clarification/Discuss Patient's RFE
-49	Other Preventive Procedures
-50	Medicat-Script/Reqst/Renew/Inject
-51	Incise/Drain/Flush/Aspirate
-52	Excise/Remove/Biopsy/Destruction/Debride
-53	Instrument/Catheter/Intubate/Dilate
-54	Repair/Fixate-Suture/Cast/Prosthetic
-55	Local Injection/Infiltration
-56	Dress/Press/Compress/Tamponade
-57	Physical Medicine/Rehabilitation
-58	Therapeutic Counselling/Listening
-59	Other Therapeutic Procedure NEC
-60	Results Tests/Procedures
-61	Results Exam/Test/Record
-62	Administrative Procedure
-63	Follow-up Encounter Unspecified
-64	Encounter Initiated by Provider
-65	Encounter Initiated third person
-66	Refer to Other Provider (EXCL. M.D.)
-67	Referral to Physician/Specialist/Clinic/Hospital
-68	Other Referrals NEC
-69	Other Reason for Encounter NEC

General and Unspecified A

A01	Pain general/multiple sites
A02	Chills
A03	Fever
A04	Weakness/tiredness general
A05	Feeling ill
A06	Fainting/syncope
A07	Coma
A08	Swelling
A09	Sweating problem
A10	Bleeding/haemorrhage NOS
A11	Chest pain NOS
A13	Concern/fear medical treatment
A16	Irritable infant
A18	Concern about appearance
A20	Euthanasia request/discussion
A21	Risk factor for malignancy
A23	Risk factor NOS
A25	Fear of death/dying
A26	Fear of cancer NOS
A27	Fear of other disease NOS
A28	Limited function/disability NOS
A29	General symptom/complaint other
A70	Tuberculosis
A71	Measles
A72	Chickenpox
A73	Malaria
A74	Rubella
A75	Infectious mononucleosis
A76	Viral exanthem other
A77	Viral disease other/NOS
A78	Infectious disease other/NOS
A79	Malignancy NOS
A80	Trauma/injury NOS
A81	Multiple trauma/injuries
A82	Secondary effect of trauma
A84	Poisoning by medical agent
A85	Adverse effect medical agent
A86	Toxic effect non-medicinal substance
A87	Complication of medical treatment
A88	Adverse effect physical factor
A89	Effect prosthetic device
A90	Congenital anomaly OS/multiple
A91	Abnormal result investigation NOS
A92	Allergy/allergic reaction NOS
A93	Premature newborn
A94	Perinatal morbidity other
A95	Perinatal mortality
A96	Death
A97	No disease
A98	Health maintenance/prevention
A99	General disease NOS

Blood, Blood Forming Organs and Immune Mechanism B

B02	Lymph gland(s) enlarged/painful
B04	Blood symptom/complaint
B25	Fear of aids/HIV
B26	Fear cancer blood/lymph
B27	Fear blood/lymph disease other
B28	Limited function/disability
B29	Sympt/complt lymph/immune other
B70	Lymphadenitis acute
B71	Lymphadenitis non-specific
B72	Hodgkin's disease/lymphoma
B73	Leukaemia
B74	Malignant neoplasm blood other
B75	Benign/unspecified neoplasm blood
B76	Ruptured spleen traumatic
B77	Injury blood/lymph/spleen other
B78	Hereditary haemolytic anaemia
B79	Congen.anom. blood/lymph other
B80	Iron deficiency anaemia
B81	Anaemia, Vitamin B12/folate def.
B82	Anaemia other/unspecified
B83	Purpura/coagulation defect
B84	Unexplained abnormal white cells
B87	Splenomegaly
B90	HIV-infection/aids
B99	Blood/lymph/spleen disease other

PROCESS CODES

SYMPTOMS/COMPLAINTS

INFECTIONS

NEOPLASMS

INJURIES

CONGENITAL ANOMALIES

OTHER DIAGNOSES

Digestive D

D01	Abdominal pain/cramps general
D02	Abdominal pain epigastric
D03	Heartburn
D04	Rectal/anal pain
D05	Perianal itching
D06	Abdominal pain localized other
D07	Dyspepsia/indigestion
D08	Flatulence/gas/belching
D09	Nausea
D10	Vomiting
D11	Diarrhoea
D12	Constipation
D13	Jaundice
D14	Haematemesis/vomiting blood
D15	Melaena
D16	Rectal bleeding
D17	Incontinence of bowel
D18	Change faeces/bowel movements
D19	Teeth/gum symptom/complaint
D20	Mouth/tongue/lip symptom/complt.
D21	Swallowing problem
D23	Hepatomegaly
D24	Abdominal mass NOS
D25	Abdominal distension
D26	Fear of cancer of digestive system
D27	Fear of digestive disease other
D28	Limited function/disability (d)
D29	Digestive symptom/complaint other
D70	Gastrointestinal infection
D71	Mumps
D72	Viral hepatitis
D73	Gastroenteritis presumed infection
D74	Malignant neoplasm stomach
D75	Malignant neoplasm colon/rectum
D76	Malignant neoplasm pancreas
D77	Malig. neoplasm digest other/NOS
D78	Neoplasm digest benign/uncertain
D79	Foreign body digestive system
D80	Injury digestive system other
D81	Congen. anomaly digestive system
D82	Teeth/gum disease
D83	Mouth/tongue/lip disease
D84	Oesophagus disease
D85	Duodenal ulcer
D86	Peptic ulcer other
D87	Stomach function disorder
D88	Appendicitis
D89	Inguinal hernia
D90	Hiatus hernia
D91	Abdominal hernia other
D92	Diverticular disease
D93	Irritable bowel syndrome
D94	Chronic enteritis/ulcerative colitis
D95	Anal fissure/perianal abscess
D96	Worms/other parasites
D97	Liver disease NOS
D98	Cholecystitis/cholelithiasis
D99	Disease digestive system, other

Eye F

F01	Eye pain
F02	Red eye
F03	Eye discharge
F04	Visual floaters/spots
F05	Visual disturbance other
F13	Eye sensation abnormal
F14	Eye movements abnormal
F15	Eye appearance abnormal
F16	Eyelid symptom/complaint
F17	Glasses symptom/complaint
F18	Contact lens symptom/complaint
F27	Fear of eye disease
F28	Limited function/disability (f)
F29	Eye symptom/complaint other
F70	Conjunctivitis infectious
F71	Conjunctivitis allergic
F72	Blepharitis/stye/chalazion
F73	Eye infection/inflammation other
F74	Neoplasm of eye/adnexa
F75	Contusion/haemorrhage eye
F76	Foreign body in eye
F79	Injury eye other
F80	Blocked lacrimal duct of infant
F81	Congenital anomaly eye other
F82	Detached retina
F83	Retinopathy
F84	Macular degeneration
F85	Corneal ulcer
F86	Trachoma
F91	Refractive error
F92	Cataract
F93	Glaucoma
F94	Blindness
F95	Strabismus
F99	Eye/adnexa disease, other

Ear H

H01	Ear pain/earache
H02	Hearing complaint
H03	Tinnitus, ringing/buzzing ear
H04	Ear discharge
H05	Bleeding ear
H13	Plugged feeling ear
H15	Concern with appearance of ears
H27	Fear of ear disease
H28	Limited function/disability ear
H29	Ear symptom/complaint other
H70	Otitis externa
H71	Acute otitis media/myringitis
H72	Serous otitis media
H73	Eustachian salpingitis
H74	Chronic otitis media
H75	Neoplasm of ear
H76	Foreign body in ear
H77	Perforation ear drum
H78	Superficial injury of ear
H79	Ear injury other
H80	Congenital anomaly of ear
H81	Excessive ear wax
H82	Vertiginous syndrome
H83	Otosclerosis
H84	Presbyacusis
H85	Acoustic trauma
H86	Deafness
H99	Ear/mastoid disease, other

Cardiovascular K

K01	Heart pain
K02	Pressure/tightness of heart
K03	Cardiovascular pain NOS
K04	Palpitations/awareness of heart
K05	Irregular heartbeat other
K06	Prominent veins
K07	Swollen ankles/oedema
K22	Risk factor cardiovascular disease
K24	Fear of heart disease
K25	Fear of hypertension
K27	Fear cardiovascular disease other
K28	Limited function/disability (k)
K29	Cardiovascular sympt./complt. other
K70	Infection of circulatory system
K71	Rheumatic fever/heart disease
K72	Neoplasm cardiovascular
K73	Congenital anomaly cardiovascular
K74	Ischaemic heart disease w. angina
K75	Acute myocardial infarction
K76	Ischaemic heart disease w/o angina
K77	Heart failure
K78	Atrial fibrillation/flutter
K79	Paroxysmal tachycardia
K80	Cardiac arrhythmia NOS
K81	Heart/arterial murmur NOS
K82	Pulmonary heart disease
K83	Heart valve disease NOS
K84	Heart disease other
K85	Elevated blood pressure
K86	Hypertension uncomplicated
K87	Hypertension complicated
K88	Postural hypotension
K89	Transient cerebral ischaemia
K90	Stroke/cerebrovascular accident
K91	Cerebrovascular disease
K92	Atherosclerosis/PVD
K93	Pulmonary embolism
K94	Phlebitis/thrombophlebitis
K95	Varicose veins of leg
K96	Haemorrhoids
K99	Cardiovascular disease other

Musculoskeletal L

L01	Neck symptom/complain
L02	Back symptom/complaint
L03	Low back symptom/complaint
L04	Chest symptom/complaint
L05	Flank/axilla symptom/complaint
L07	Jaw symptom/complaint
L08	Shoulder symptom/complaint
L09	Arm symptom/complaint
L10	Elbow symptom/complaint
L11	Wrist symptom/complaint
L12	Hand/finger symptom/complaint
L13	Hip symptom/complaint
L14	Leg/thigh symptom/complaint
L15	Knee symptom/complaint
L16	Ankle symptom/complaint
L17	Foot/toe symptom/complaint
L18	Muscle pain
L19	Muscle symptom/complaint NOS
L20	Joint symptom/complaint NOS
L26	Fear of cancer musculoskeletal
L27	Fear musculoskeletal disease other
L28	Limited function/disability (l)
L29	Sympt/complt. Musculoskeletal other
L70	Infections musculoskeletal system
L71	Malignant neoplasm musculoskeletal
L72	Fracture: radius/ulna
L73	Fracture: tibia/fibula
L74	Fracture: hand/foot bone
L75	Fracture: femur
L76	Fracture: other
L77	Sprain/strain of ankle
L78	Sprain/strain of knee
L79	Sprain/strain of joint NOS
L80	Dislocation/subluxation
L81	Injury musculoskeletal NOS
L82	Congenital anomaly musculoskeletal
L83	Neck syndrome
L84	Back syndrome w/o radiating pain
L85	Acquired deformity of spine
L86	Back syndrome with radiating pain
L87	Bursitis/tendinitis/synovitis NOS
L88	Rheumatoid/seropositive arthritis
L89	Osteoarthrosis of hip
L90	Osteoarthrosis of knee
L91	Osteoarthrosis other
L92	Shoulder syndrome
L93	Tennis elbow
L94	Osteochondrosis
L95	Osteoporosis
L96	Acute internal damage knee
L97	Neoplasm benign/unspec musculo.
L98	Acquired deformity of limb
L99	Musculoskeletal disease, other

Neurological N

N01	Headache
N03	Pain face
N04	Restless legs
N05	Tingling fingers/feet/toes
N06	Sensation disturbance other
N07	Convulsion/seizure
N08	Abnormal involuntary movements
N16	Disturbance of smell/taste
N17	Vertigo/dizziness
N18	Paralysis/weakness
N19	Speech disorder
N26	Fear cancer neurological system
N27	Fear of neurological disease other
N28	Limited function/disability (n)
N29	Neurological symptom/complt. other
N70	Poliomyelitis
N71	Meningitis/encephalitis
N72	Tetanus
N73	Neurological infection other
N74	Malignant neoplasm nervous system
N75	Benign neoplasm nervous system
N76	Neoplasm nervous system unspec.
N79	Concussion
N80	Head injury other
N81	Injury nervous system other
N85	Congenital anomaly neurological
N86	Multiple sclerosis
N87	Parkinsonism
N88	Epilepsy
N89	Migraine
N90	Cluster headache
N91	Facial paralysis/bell's palsy
N92	Trigeminal neuralgia
N93	Carpal tunnel syndrome
N94	Peripheral neuritis/neuropathy
N95	Tension headache
N99	Neurological disease, other

Psychological P

P01 Feeling anxious/nervous/tense
P02 Acute stress reaction
P03 Feeling depressed
P04 Feeling/behaving irritable/angry
P05 Senility, feeling/behaving old
P06 Sleep disturbance
P07 Sexual desire reduced
P08 Sexual fulfilment reduced
P09 Sexual preference concern
P10 Stammering/stuttering/tic
P11 Eating problem in child
P12 Bedwetting/enuresis
P13 Encopresis/bowel training problem
P15 Chronic alcohol abuse
P16 Acute alcohol abuse
P17 Tobacco abuse
P18 Medication abuse
P19 Drug abuse
P20 Memory disturbance
P22 Child behaviour symptom/complaint
P23 Adolescent behav. Symptom/complt.
P24 Specific learning problem
P25 Phase of life problem adult
P27 Fear of mental disorder
P28 Limited function/disability (p)
P29 Psychological symptom/complt other
P70 Dementia
P71 Organic psychosis other
P72 Schizophrenia
P73 Affective psychosis
P74 Anxiety disorder/anxiety state
P75 Somatization disorder
P76 Depressive disorder
P77 Suicide/suicide attempt
P78 Neuraesthenia/surmenage
P79 Phobia/compulsive disorder
P80 Personality disorder
P81 Hyperkinetic disorder
P82 Post-traumatic stress disorder
P85 Mental retardation
P86 Anorexia nervosa/bulimia
P98 Psychosis NOS/other
P99 Psychological disorders, other

Respiratory R

R01 Pain respiratory system
R02 Shortness of breath/dyspnoea
R03 Wheezing
R04 Breathing problem, other
R05 Cough
R06 Nose bleed/epistaxis
R07 Sneezing/nasal congestion
R08 Nose symptom/complaint other
R09 Sinus symptom/complaint
R21 Throat symptom/complaint
R23 Voice symptom/complaint
R24 Haemoptysis
R25 Sputum/phlegm abnormal
R26 Fear of cancer respiratory system
R27 Fear of respiratory disease, other
R28 Limited function/disability (r)
R29 Respiratory symptom/complaint oth.
R71 Whooping cough
R72 Strep throat
R73 Boil/abscess nose
R74 Upper respiratory infection acute
R75 Sinusitis acute/chronic
R76 Tonsillitis acute
R77 Laryngitis/tracheitis acute
R78 Acute bronchitis/bronchiolitis
R79 Chronic bronchitis
R80 Influenza
R81 Pneumonia
R82 Pleurisy/pleural effusion
R83 Respiratory infection other
R84 Malignant neoplasm bronchus/lung
R85 Malinant neoplasm respiratory, other
R86 Benign neoplasm respiratory
R87 Foreign body nose/larynx/bronch
R88 Injury respiratory other
R89 Congenital anomaly respiratory
R90 Hypertrophy tonsils/adenoids
R92 Neoplasm respiratory unspecified
R95 Chronic obstructive pulmonary dis
R96 Asthma
R97 Allergic rhinitis
R98 Hyperventilation syndrome
R99 Respiratory disease other

PROCESS CODES
SYMPTOMS/COMPLAINTS
INFECTIONS
NEOPLASMS
INJURIES
CONGENITAL ANOMALIES
OTHER DIAGNOSES

Skin S

S01 Pain/tenderness of skin
S02 Pruritus
S03 Warts
S04 Lump/swelling localized
S05 Lumps/swellings generalized
S06 Rash localized
S07 Rash generalized
S08 Skin colour change
S09 Infected finger/toe
S10 Boil/carbuncle
S11 Skin infection post-traumatic
S12 Insect bite/sting
S13 Animal/human bite
S14 Burn/scald
S15 Foreign body in skin
S16 Bruise/contusion
S17 Abrasion/scratch/blister
S18 Laceration/cut
S19 Skin injury other
S20 Corn/callosity
S21 Skin texture symptom/complaint
S22 Nail symptom/complaint
S23 Hair loss/baldness
S24 Hair/scalp symptom/complaint
S26 Fear of cancer of skin
S27 Fear of skin disease other
S28 Limited function/disability (s)
S29 Skin symptom/complaint other
S70 Herpes zoster
S71 Herpes simplex
S72 Scabies/other acariasis
S73 Pediculosis/skin infestation other
S74 Dermatophytosis
S75 Moniliasis/candidiasis skin
S76 Skin infection other
S77 Malignant neoplasm of skin
S78 Lipoma
S79 Neoplasm skin benign/unspecified
S80 Solar keratosis/sunburn
S81 Haemangioma/lymphangioma
S82 Naevus/mole
S83 Congenital skin anomaly other
S84 Impetigo
S85 Pilonidal cyst/fistula
S86 Dermatitis seborrhoeic
S87 Dermatitis/atopic eczema
S88 Dermatitis contact/allergic
S89 Diaper rash
S90 Pityriasis rosea
S91 Psoriasis
S92 Sweat gland disease
S93 Sebaceous cyst
S94 Ingrowing nail
S95 Molluscum contagiosum
S96 Acne
S97 Chronic ulcer skin
S98 Urticaria
S99 Skin disease, other

Endocrine/Metabolic and Nutritional T

T01 Excessive thirst
T02 Excessive appetite
T03 Loss of appetite
T04 Feeding problem of infant/child
T05 Feeding problem of adult
T07 Weight gain
T08 Weight loss
T10 Growth delay
T11 Dehydration
T26 Fear of cancer of endocrine system
T27 Fear endocrine/metabolic dis other
T28 Limited function/disability (t)
T29 Endocrine/met./sympt/complt other
T70 Endocrine infection
T71 Malignant neoplasm thyroid
T72 Benign neoplasm thyroid
T73 Neoplasm endocrine oth/unspecified
T78 Thyroglossal duct/cyst
T80 Congenital anom endocrine/metab.
T81 Goitre
T82 Obesity
T83 Overweight
T85 Hyperthyroidism/thyrotoxicosis
T86 Hypothyroidism/myxoedema
T87 Hypoglycaemia
T89 Diabetes insulin dependent
T90 Diabetes non-insulin dependent
T91 Vitamin/nutritional deficiency
T92 Gout
T93 Lipid disorder
T99 Endocrine/metab/nutrit. dis. other

Urological U

U01 Dysuria/painful urination
U02 Urinary frequency/urgency
U04 Incontinence urine
U05 Urination problems other
U06 Haematuria
U07 Urine symptom/complaint other
U08 Urinary retention
U13 Bladder symptom/complaint other
U14 Kidney symptom/complaint
U26 Fear of cancer of urinary system
U27 Fear of urinary disease other
U28 Limited function/disability urinary
U29 Urinary symptom/complaint other
U70 Pyelonephritis/pyelitis
U71 Cystitis/urinary infection other
U72 Urethritis
U75 Malignant neoplasm of kidney
U76 Malignant neoplasm of bladder
U77 Malignant neoplasm urinary other
U78 Benign neoplasm urinary tract
U79 Neoplasm urinary tract NOS
U80 Injury urinary tract
U85 Congenital anomaly urinary tract
U88 Glomerulonephritis/nephrosis
U90 Orthostatic albumin./proteinuria
U95 Urinary calculus
U98 Abnormal urine test NOS
U99 Urinary disease, other

Pregnancy, Childbearing, Family Planning W

W01 Question of pregnancy
W02 Fear of pregnancy
W03 Antepartum bleeding
W05 Pregnancy vomiting/nausea
W10 Contraception postcoital
W11 Contraception oral
W12 Contraception intrauterine
W13 Sterilization
W14 Contraception other
W15 Infertility/subfertility
W17 Post-partum bleeding
W18 Post-partum symptom/complaint oth.
W19 Breast/lactation symptom/complaint
W21 Concern body image in pregnancy
W27 Fear complications of pregnancy
W28 Limited function/disability (w)
W29 Pregnancy symptom/complaint other
W70 Puerperal infection/sepsis
W71 Infection complicating pregnancy
W72 Malignant neoplasm relate to preg.
W73 Benign/unspec. neoplasm/pregnancy
W75 Injury complicating pregnancy
W76 Congenital anomaly complicate preg.
W78 Pregnancy
W79 Unwanted pregnancy
W80 Ectopic pregnancy
W81 Toxaemia of pregnancy
W82 Abortion spontaneous
W83 Abortion induced
W84 Pregnancy high risk
W85 Gestational diabetes
W90 Uncomplicate labour/delivery live
W91 Uncomplicate labour/delivery still
W92 Complicate labour/ delivery livebirth
W93 Complicate labour/delivery stillbirth
W94 Puerperal mastitis
W95 Breast disorder in pregnancy other
W96 Complications of puerperium other
W99 Disorder pregnancy/delivery, other

Female Genital X

X01 Genital pain female
X02 Menstrual pain
X03 Intermenstrual pain
X04 Painful intercourse female
X05 Menstruation absent/scanty
X06 Menstruation excessive
X07 Menstruation irregular/frequent
X08 Intermenstrual bleeding
X09 Premenstrual symptom/complaint
X10 Postponement of menstruation
X11 Menopausal symptom/complaint
X12 Postmenopausal bleeding
X13 Postcoital bleeding
X14 Vaginal discharge
X15 Vaginal symptom/complaint other
X16 Vulval symptom/complaint
X17 Pelvis symptom/complaint female
X18 Breast pain female
X19 Breast lump/mass female
X20 Nipple symptom/complaint female
X21 Breast symptom/complt. female other
X22 Concern breast appearance female
X23 Fear sexually transmitted disease (f)
X24 Fear of sexual dysfunction female
X25 Fear of genital cancer female
X26 Fear of breast cancer female
X27 Fear genital/breast disease other (f)
X28 Limited function/disability (x)
X29 Genital symptom/complt female oth.
X70 Syphilis female
X71 Gonorrhoea female
X72 Genital candidiasis female
X73 Genital trichomoniasis female
X74 Pelvic inflammatory disease
X75 Malignant neoplasm cervix
X76 Malignant neoplasm breast female
X77 Malignant neoplasm genital other (f)
X78 Fibromyoma uterus
X79 Benign neoplasm breast female
X80 Benign neoplasm female genital
X81 Genital neoplasm oth/unspecied (f)
X82 Injury genital female
X83 Congenital anomaly genital female
X84 Vaginitis/vulvitis NOS
X85 Cervical disease NOS
X86 Abnormal cervix smear
X87 Uterovaginal prolapse
X88 Fibrocystic disease breast
X89 Premenstrual tension syndrome
X90 Genital herpes female
X91 Condylomata acuminata female
X92 Chlamydia infection genital (f)
X99 Genital disease female, other

Male Genital Y

Y01 Pain in penis
Y02 Pain in testis/scrotum
Y03 Urethral discharge
Y04 Penis symptom/complaint other
Y05 Scrotum/testis sympt/complt. other
Y06 Prostate symptom/complaint
Y07 Impotence NOS
Y08 Sexual function sympt./complt.(m)
Y10 Infertility/subfertility male
Y13 Sterilization male
Y14 Family planning male other
Y16 Breast symptom/complaint male
Y24 Fear of sexual dysfunction male
Y25 Fear sexually transmitted dis. male
Y26 Fear of genital cancer male
Y27 Fear of genital disease male other
Y28 Limited function/disability (y)
Y29 Genital sympt./complt.male other
Y70 Syphilis male
Y71 Gonorrhoea male
Y72 Genital herpes male
Y73 Prostatitis/seminal vesiculitis
Y74 Orchitis/epididymitis
Y75 Balanitis
Y76 Condylomata acuminata male
Y77 Malignant neoplasm prostate
Y78 Malign neoplasm male genital other
Y79 Benign/unspec. neoplasm gen. (m)
Y80 Injury male genital
Y81 Phimosis/redundant prepuce
Y82 Hypospadias
Y83 Undescended testicle
Y84 Congenital genl anomaly (m) other
Y85 Benign prostatic hypertrophy
Y86 Hydrocoele
Y99 Genital disease male, other

Social Problems Z

Z01 Poverty/financial problem
Z02 Food/water problem
Z03 Housing/neighbourhood problem
Z04 Social cultural problem
Z05 Work problem
Z06 Unemployment problem
Z07 Education problem
Z08 Social welfare problem
Z09 Legal problem
Z10 Health care system problem
Z11 Compliance/being ill problem
Z12 Relationship problem with partner
Z13 Partner's behaviour problem
Z14 Partner illness problem
Z15 Loss/death of partner problem
Z16 Relationship problem with child
Z18 Illness problem with child
Z19 Loss/death of child problem
Z20 Relationship prob. parent/family
Z21 Behaviour problem parent/family
Z22 Illness problem parent/family
Z23 Loss/death parent/family member
Z24 Relationship problem friend
Z25 Assault/harmful event problem
Z27 Fear of a social problem
Z28 Limited function/disability (z)
Z29 Social problem NOS

Abbreviations

Anom	anomaly
behav.	behaviour
bronch.	bronchus
complicat.	complication
congen.	congenital
dis.	disease
eval.	evaluation
exam.	examination
gen.	genital
malig.	malignant
metab.	metabolic
musculo.	musculoskeletal
NEC	not elsewhere classified
NOS	not otherwise specified
nutrit.	nutrition
oth	other
preg.	pregnancy
prob.	problem
RFE	reason for encounter
sympt.	symptom
unspec.	unspecified
w	with
w/o	without
